# The League of Mercy

**Published:** 1911-11-04

**Authors:** 


### The League of Mercy.

### GRANTS TO EXTRA-METROPOLITAN HOSPITALS.

With the sanction of Prince Alexander of Teck, the Grand President of the League of Mercy, grants for 1911, amounting in all to £2,200, have been made to extra-Metropolitan hospitals by the League. The total amount to date handed over to King Edward's Hospital Fund for London is now £153,000, in addition to which £9,861 has been given to extra-Metropolitan hospitals in counties collecting for the League. The grants to extra-Metropolitan hospitals are as follows :—

Bedfordshire.—Bute Hospital, £50.

Berkshire.—Royal Berkshire Hospital, Reading, £200; King Edward VII. Hospital and Dispensary, Windsor, £30; Faringdon Cottage Hospital, £10.

Buckinghamshire.—Northampton General Hospital, £50; Royal Bucks Hospital, Aylesbury, £30; Iver, Langley, and Denham Cottage Hospital, £5; Chesham Cottage Hospital, £5; Chalfont St. Peter's Cottage Hospital, £5; Marlow Cottage Hospital, £15.

Essex.—Essex County Hospital, Colchester, £35; Buckhurst Hill Medical and Surgical Home, £10; Clacton and District Hospital, £5; Chelmsford and Essex Hospital and Dispensary, £45; Brentwood District Cottage Hospital, £10; Victoria Cottage Hospital, Romford, £15; Victoria Hospital, Southend, £20.

Hampshire.—Royal Portsmouth, Portsea, and Gosport Hospital, £150; Royal South Hants and Southampton Hospital, £150; Royal Hants County Hospital, £100; Royal Isle of Wight Infirmary and County Hospital, £5; Fleet Cottage Hospital, £5; Andover Cottage Hospital, £10; Inwood Cottage Hospital, Alton, £5.

Hertfordshire.—Hertford County Hospital, £50; West Herts Infirmary, Hemel Hempstead, £20; Watford District Hospital, £10; Bushey Heath Cottage Hospital, £5.

Kent.—General Kent and Canterbury Hospital, £40; Ashford Cottage Hospital, £15; Bexley Cottage Hospital, £10; Faversham Cottage Hospital, £5; Gravesend Hospital, £30; Royal Victoria Hospital, Folkestone, £15; Victoria Home for Invalid Children, Margate, £10; Queen Victoria Memorial Cottage Hospital, Herne Bay, £5; West Kent General Hospital, Maidstone, £35; Kent County Ophthalmic Hospital, Maidstone, £20; Children's Hip Hospital, Sevenoaks, £20; Chislehurst and Cray Cottage Hospital, £10; Bromley Cottage Hospital, £15; Phillips Memorial Homœopathic Hospital, £10; Tunbridge Wells

General Hospital, £40; Tunbridge Wells Homœopathic Hospital, £10.

Middlesex.—St. John's Hospital, Twickenham, £10; Hounslow Hospital, £20; Hanwell Cottage Hospital, £10; Enfield Cottage Hospital, £10; Teddington Cottage Hospital, £10; Hayes Cottage Hospital, £5.

Oxfordshire.—Radcliffe Infirmary and County Hospital, Oxford, £150; Horton Infirmary, Banbury, £30; Oxford Eye Hospital, £20; Watlington Cottage Hospital, £5.

Suffolk.—East Suffolk and Ipswich Hospital, £50; West Suffolk General Hospital, Bury St. Edmunds, £50; St. Leonards Hospital, Sudbury, £10.

Surrey.—Egham Cottage Hospital, £10; Victoria Cottage Hospital, Woking, £10; Croydon General Hospital, £20; Sutton Hospital, £10; Caterham Cottage Hospital, £10; Epsom and Ewell Cottage Hospital, £10; Carshalton District Cottage Hospital, £10; Walton-on-Thames, Hersham, and Oatlands Cottage Hospital, £5; Victoria Memorial Cottage Hospital, Leatherhead, £5; Thames Ditton Cottage Hospital, £5; East and West Molesey and Hampton Court Cottage Hospital, £5; Royal Surrey County Hospital, Guildford, £75; Victoria Hospital, Kingston, £10; Surbiton Cottage Hospital, £10.

Sussex.—Royal Alexandra Hospital for Children, Brighton, £25; Hastings, St. Leonards, and East Sussex Hospital, £150; Buchanan Hospital, St. Leonards, £10; All Saints Convalescent Home, St. Leonards, £10; Princess Alice Memorial Hospital, Eastbourne, £20; Leaf Homœopathic Hospital, Eastbourne, £10; Queen Victoria Cottage Hospital, East Grinstead, £10; Eliot Cottage Hospital, Haywards Heath, £10; Horsham Cottage Hospital, £10; Petworth Cottage Hospital, £5; Lewes Dispensary and Infirmary and Victoria Hospital, £10; Crowborough District Nursing Association, £10; Uckfield Cottage Hospital, £5.

